# A micromechanical muscle model for determining the impact of motor unit fiber clustering on force transmission in aging skeletal muscle

**DOI:** 10.1007/s10237-019-01152-2

**Published:** 2019-05-02

**Authors:** Aron Teklemariam, Emma Hodson-Tole, Neil D. Reeves, Glen Cooper

**Affiliations:** 1 School of Engineering, Manchester Metropolitan University, Manchester, UK; 2 Research Centre for Musculoskeletal Science and Sports Medicine, School of Healthcare Science, Manchester Metropolitan University, Manchester, UK; 3 School of Engineering, University of Manchester, Manchester, M13 9PL UK

**Keywords:** Muscle, Finite element modeling, Spacial activation patterns, Motor unit, Fiber clustering, Force

## Abstract

This study used a micromechanical finite element muscle model to investigate the effects of the redistribution of spatial activation patterns in young and old muscle. The geometry consisted of a bundle of 19 active muscle fibers encased in endomysium sheets, surrounded by passive tissue to model a fascicle. Force was induced by activating combinations of the 19 active muscle fibers. The spacial clustering of muscle fibers modeled in this study showed unbalanced strains suggesting tissue damage at higher strain levels may occur during higher levels of activation and/or during dynamic conditions. These patterns of motor unit remodeling are one of the consequences of motor unit loss and reinnervation associated with aging. The results did not reveal evident quantitative changes in force transmission between old and young adults, but the patterns of stress and strain distribution were affected, suggesting an uneven distribution of the forces may occur within the fascicle that could provide a mechanism for muscle injury in older muscle.

## Introduction

Skeletal muscles produce the forces required to maintain body posture and complete the myriad of activities associated with daily living. The ability of muscles to produce force relies not only on the activation level but also on properties related to their structure (e.g., fiber length, pennation angle, number of fibers, physiological cross-sectional area) and the connective materials into which the fibers are embedded to form the muscle macrostructure. During aging muscle properties change, as do their ability to produce force. The decline in skeletal muscle strength has been related to the quality of force per unit area of the muscle tissue related to factors such as: fat infiltration (Rahemi et al. 2015), lateral force transmission between fibers through the endomysium (Ramaswamy et al. 2011; Zhang and Gao 2014), and fiber type (rather than quantity of fibers (Goodpaster et al. 2006). The underlying characteristics of reduced muscle quality are poorly defined, limiting attempts to understand mechanisms underpinning loss of function with age. If the muscle is considered as a fiber-reinforced composite material (Huijing 1999), where the matrix is the connective tissue, the problem can be split into intrinsic and extrinsic factors. Intrinsic factors refer to alterations in the mechanical properties of the fibers and the matrix tissue (Gao et al. 2008; Ward et al. 2009; Plate et al. 2013; Wood et al. 1985; Wang et al. 2014). Extrinsic factors can refer to alterations in the proportion of the matrix relative to the fibers (Smith and Barton 2014), length of the fibers and the spatial distribution of muscle fibers from the same motor unit (Faulkner et al. 2007; Kadhiresan et al. 1996).

When considering intrinsic factors influencing force production of aging muscle, it is well established that there is a change in fiber type proportions, favouring an increase in slower types and reducing mechanical power producing capabilities (Lexell 1995; Lexell et al. 1988). In addition, the mechanical properties (stiffness) of connective tissues, such as collagen, are important for transmission of forces from the myofibers through the muscle belly (Purslow and Trotter 1994; Trotter et al. 1995), Street’s (1983) experiment on myofibers at the myotendinous junction revealed the existence of this force transmission which is believed to be caused by costameric proteins that link the muscle proteins (actin) to the collagen fibers outside the sarcolemma, specifically in the first layer, the basal lamina (Bloch and Gonzalez-Serratos 2003; Peter et al. 2011; Monti et al. 1999). The possible structural mechanism for force transmission is well explained by Purslow (2002) who showed how the stretch of the myofibers will pull the collagen fibrils that are connected to other myofibers. Consequently shear stress between myofibers is mediated by the surrounding collagen structures. This transmission is not direct, but a connection mediated by the collagen layers which reflects the role of the specific layer. Between myofibers, the collagen pattern is called the endomysium and its role is to accommodate the fibers length change while distributing the stress transversally (Purslow and Trotter 1994; Trotter and Purslow 1992). The collagen pattern surrounding the bundle of myofibers (or fascicle) is called the perimysium, and its role is to accommodate shape changes of the fascicles, allowing smooth deformations (Purslow 1999). These functional roles can be deduced from the pattern of collage fibers. For instance, the orientation of the collagen fibers of the endomysium differs from the perimysium and changes during the muscle contraction to accommodate the length change of the myofibers. In addition, the perimysium has three layers of collagen, each one with different characteristics. Despite the differences and characteristics, these tissues share a general common pattern that consists of collagen fibers and an amorphous matrix of hydrated proteoglycans which contribute to mechanically link the collagen fiber network (Purslow 2010). The properties of the endomysium and perimysium also change with age (Zhang and Gao 2014; Gao et al. 2008). For example, degeneration of collagen, believed to be associated with an increase of cross-linking within the collagen fibrils related with the reduced fibrils turnover (Gao et al. 2008; Rodrigues et al. 1996; Kjaer 2004), is linked to increased muscle stiffness (Galeski et al. 1977) and is linked to a reduction in lateral force transmission (Kadhiresan et al. 1996).

When considering possible extrinsic factors influencing force production, several factors may be influencial. For example, due to aging fat infiltration and fibrosis commonly occurs (Smith and Barton 2014; Kent-Braun et al. 1985) and decreases in some fiber diameters may occur (Lexell 1995), both of which may significantly alter the volume fraction (volume of fibers/volume of matrix) within the muscle. This may not, however, in itself alter all force producing capabilities, as a simulation study by Sharafi and Blemker (2011)showed that force transmission was not influenced by volume fraction or changes in the amount of collagen in the muscle. However, it may be that the distribution of these additional materials through the muscle has a significant impact. For example, a simulation study by Rahemi et al. (2015) showed that the higher stiffness of models that included intramuscular fat resulted in the prediction of lower fiber stress and that the results were affected by whether the fat was modeled as a clump or dispersed through the muscle tissue. The arrangement of matrix base material and fibers through the muscle therefore has an effect on force transmission. While Rahemi et al. (2015) manipulated the proportion and distribution of fat, it is unclear whether the spatial arrangement of muscle fibers within the matrix may also influence force transmission and this could be a further important factor when considering aging muscle.

Muscle fibers belonging to the same motor units are typically expected to be randomly distributed within a muscle region (Monti et al. 2001). However, there is evidence that in some muscles (e.g., human medial gastrocnemius) fibers within the same motor unit are spatially localized within a relatively small area (Hodson-Tole et al. 2012; Vieira et al. 2016). Clustering of fibers from the same unit is also likely to occur in aged muscle, when de-innervated fibers are reinnervated through collateral axonal sprouting of nearby units (Kadhiresan et al. 1996). Consequently, changes in force transmission due to aging could be caused by a change in the distribution of the activated fibers. To the author’s knowledge, the consequence of this phenomenon has not previously been investigated either experimentally or through modeling. Therefore, this work uses a micromechanical muscle model to investigate the effects of redistribution of the fibers belonging to the same motor unit to simulate the reinnervation of fibers in aged muscle. Specific patterns of activation within the fascicle are unknown, so for the purposes of this study activation levels of approximately 20% and 40% will be used to investigate a range of different activation patterns and its effect on force transmission and stress distribution in the tissue. The hypothesis is that a clustered fiber distribution creates excessive stress/strain concentrations within the fascicle and in the tissue surrounding the fascicle.

## Method

A finite element model implemented in Comsol Multiphysics software (version 5.0, Cambridge, UK) is used to investigate the force production and the interaction between the fibers and endomysium within a fascicle, the surrounding tissue and the extended tissue (definitions given in Table 1).

**Table 1:** Definitions of the tissues

Tissue	Definition
Fascicle	A group of muscle fibers each wrapped by an endomysium sheet and joined together in a bundle
Extended tissue	A continuation of the fascicle, but with tissue properties that reflects the stiffness of the muscular–tendon junction
Surrounding tissue	The muscle tissue that surround the fascicle and that will be compressed as the fascicle contracts/expands

### Model geometry

**Fig. 1:**
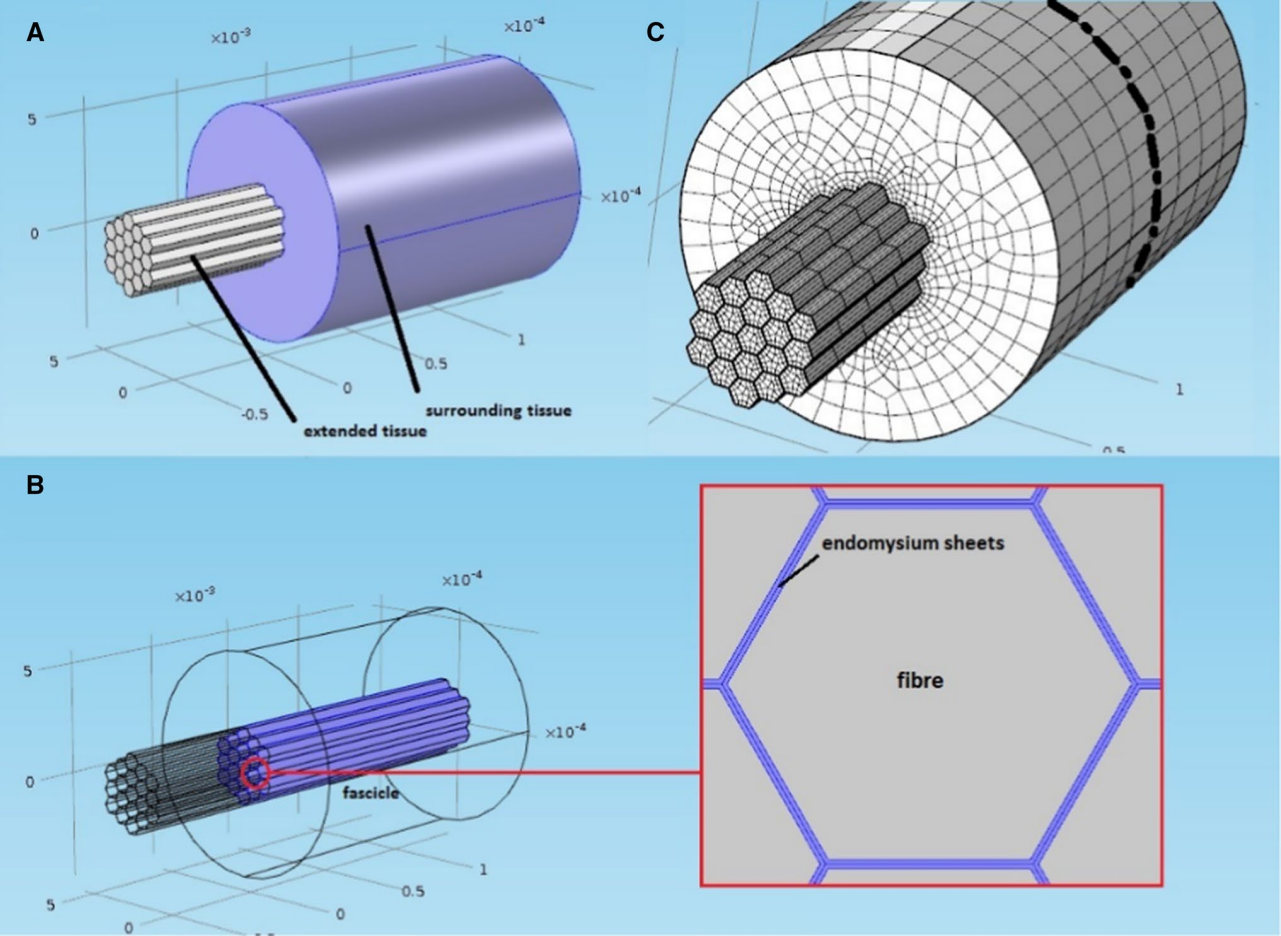
Geometry and finite element (FE) model of the muscle tissue. **a** Geometry of the tissue containing three distinct regions (defined in Table 1) the fascicle, the sounding tissue and the extended tissue. **b** The fascicle is inside the surrounding tissue and is comprised of 19 muscle fibers each of which is wrapped in an endomysium sheet. **c** Finite element model of the tissue showing dashed line where the model was cut to obtain axial stress values at this section for analysis

Figure 1a, b shows the geometry of the model, which consisted of a fascicle made of 19 muscle fibers each wrapped by an endomysial sheet. The fascicle is wrapped in a perimysial sheet and is enclosed by the surrounding tissue that makes up the belly of the muscle. The number of fibers in a fascicle can vary considerably but as a balance between managing the complexity of the model and accurately mimicking fiber numbers within a fascicle, 19 fibers was deemed to be an appropriate number for the purposes of modeling a simple fascicle. The dimensions for the fibers and the endomysium were taken from work by Sharafi and Blemker (2010). Structures surrounding the fascicle are described as a homogeneous tissue block shown in Fig. 1a, b. A description of the tissues involved in the model and the terminology used is reported in Table 1.

### Constitutive properties of the skeletal muscle model

An elastic material that undergoes deformation retains energy, strain energy, that is expressed as a scalar function of the deformation. Here, the strain energy functions used to describe the muscle tissue mechanical behavior were obtained from mechanical tensile/compressive tests performed on animal muscle tissue reported in the literature (Bosboom et al. 2001; Chi et al. 2010; Prado et al. 2005). As a recent study confirmed that skeletal muscle properties follow a pattern of aging that is qualitatively consistent across species (i.e., humans and rats) (Ballak et al. 2014), these values can inform understanding of aging in human muscle and were hence used here. The results were fitted to hyperelastic models (Mooney 1940; Ogden 1972; Yeoh 1993), using the process described below (Table 2).

**Table 2:** Muscle constitutive properties parameters: the strain energy function Mooney–Rivlin coefficients, the bulk property and the muscle density taken from Chi et al. (2010)

Mooney–Rivlin coefficients	Values used
$C_{10}$	64,300 Pa
$C_{01}$	− 38,000 Pa
$C_{11}$	− 43 Pa
$C_{20}$	5400 Pa
$C_{02}$	5 Pa
$K$ (bulk modulus)	$5 \times 10^{7}$ Pa
*ρ* (density)	1000 kg/m^3^

The strain energy was derived from the stretch invariants that are invariant to both the coordinate system and the strain measure (Mase et al. 2010). For ease of computation, the Cauchy Green right strain tensor,$C$, was used to calculate the stretch invariants (Crisfield 1997). Therefore, the hyperelastic constitutive models used to describe the fiber and the endomysium tissues were defined through strain energy functions, $W_{\text{s}}$, which are a function of the $C_{ij}$ Cauchy-Green tensor’s invariants, $I_{i }$. Once these are defined, the second Piola–Kirchhoff stress tensor, $S_{ij}$, is computed, shown in Eq. 1.

1 $$S_{ij} = 2\frac{{\partial W_{\text{s}} }}{{\partial C_{ij} }}.$$

This tensor has no direct physical meaning but has the advantage of being symmetric and invariant to rotations. It represents the current stress in the original configuration, meaning that it also tracks the fiber direction. Due to this property the myofiber stress components were added in the Second Piola–Kirchhoff stress tensor, to the component of the tensor along the fiber direction so that the stress would align to the fibers path as the muscle contracts.

As the fascicle was defined as a nearly incompressible material, the strain energy was divided into isochoric (deformation) and volumetric (volume change) components (Rahemi et al. 2014; Blemker et al. 2005), shown in Eq. 2.

2 $$W_{\text{s}} = W_{\text{iso}} + W_{\text{vol}} .$$

The volumetric strain energy will induce a volumetric stress (pressure), $p_{\text{p}}$, shown in Eq. 3.

3 $$p_{\text{p}} = - \frac{{\partial W_{\text{vol}} }}{\partial J} = - k\left( {J - 1} \right).$$

As a result, the total contribution to the second Piola–Kirchhoff stress will be given by Eq. 4.

4 $$S_{ij} = - p_{\text{p}} JC^{ - 1} + 2\frac{{\partial W_{\text{iso}} }}{{\partial C_{ij} }}.$$

For the myofiber specific case, the second Piola–Kirchhoff stress along the axial (fiber) direction was expressed in Eq. 5.

5 $$S_{\text{axial}} = - p_{\text{p}} JC^{ - 1} + 2\frac{{\partial W_{\text{base}} }}{{\partial C_{ij} }} + S_{\text{active}} .$$

The Cauchy stress, *σ*, is computed afterward through the relationship with $S_{ij}$:shown in Eq. 6 and the second Piola–Kirchhoff stress given in Eq. 4.

6 $$\sigma = J^{ - 1} FSF^{T} = - p_{\text{p}} I + 2J^{ - 1} F\frac{{\partial W_{\text{iso}} }}{\partial C}F^{T} .$$

The Cauchy stress is the actual stress in the current configuration, meaning the real physical stress after the body undergoes deformation. For this reason, this is the stress tensor of interest.

The lateral force transmission is given by the collagen fibers which are pulled as the muscle contracts. At a bigger tissue scale, this can be seen as the endomysium undergoing shear strains, and transferring the muscle axial force by shear stresses. Therefore, the endomysium can be modeled as a tissue layer in between the muscle fibers. The endomysium sheets surrounding the fibers can be considered to behave in a similar manner to rubber like materials (i.e., nonlinear large deformations) (Gillies and Lieber 2011). A hyperelastic model would therefore generally be appropriate to describe the mechanical contribution of the endomysium. In the presented model the connective tissue strain energy, $W_{\text{base}}$, was described with a Mooney–Rivlin strain energy (Mooney 1940) with five coefficients calibrated from experimental data reported in the literature (Chi et al. 2010).

The stress–strain relationship of fibers within the fascicle were modeled in three parts, consisting of the fiber base material (hyperelastic behavior for collagen and fibrils in the sarcolemma), the fiber passive material (elastic behavior for titin) and activation stress (from active muscle behavior). These three stress components were summed together to produce the total stress in the fiber.

The fiber base material (extramyofibrillar elements) was modeled with a Yeoh strain energy function (Table 3) (Yeoh 1993; Rahemi et al. 2014) The fiber passive behavior [including a contribution from titin (Herzog et al. 2015)] was modeled as a nonlinear elastic material by fitting a second-order polynomial, $\sigma$, shown in Eq. 7, to experimental results from soleus fibers (Prado et al. 2005).

**Table 3:** Yeoh model parameters, taken from Rahemi et al. (2014)

Coefficients	Values used (Pa)
$c_{1}$	6750
$c_{2}$	0.0278
$c_{3}$	− 0.001975

7 $$\sigma = 176340\varepsilon^{2} - 24703\varepsilon + 505.13,$$

As the fiber direction is known and does not change as the fiber contracts, it is possible to track the axial strain directly, which means that the current stress is directly derived knowing the fibers axial deformation.

A component representing the muscle’s tendon was included in the model by extending axially the fascicle structure beyond the surrounding muscle tissue shown in Fig. 1a. This tendon was modeled as a linear elastic material and referred to as extended tissue. Stiffness values were based on data from the Achilles tendon of young and middle aged rats (1.390 ± 0.138 and 1.984 ± 0.146 N/mm, respectively) reported by Plate et al. (2013). The rats were aged 8 months (young) and 24 months (middle aged) old, which corresponds to 18 and 52 human years assuming that 13.7 rat days is equivalent to 1 human year (Ballak et al. 2014). Two Young’s Moduli, E, were derived from these stiffness values from linear elastic theory, shown in Eq. 8:

8 $$E = {\text{Young's}}\;{\text{Modulus}} = \frac{KL}{A},$$

where $K$ is the stiffness, $L$ is the length of the tissue considered (half of the fiber length) and $A$ is the total cross-sectional area of the fascicle.

The fascicle was surrounded by other contractile and connective tissue shown in Fig. 1a, b, described by an Ogden hyperelastic model (Bosboom et al. 2001; Ogden 1972) described in Eq. 9.

9 $$W\left( {\lambda_{1} ,\lambda_{2} ,\lambda_{3} } \right) = \mathop \sum \limits_{p = 1}^{N} \frac{{\mu_{p} }}{{\alpha_{p} }}\left( {\lambda_{1}^{{\alpha_{p} }} + \lambda_{2}^{{\alpha_{p} }} + \lambda_{3}^{{\alpha_{p} }} - 3} \right).$$

This strain energy is a function of the principal stretches. The parameters were fitted using in vivo experimental compressive test data ($\mu_{p} = 15.6\, {\text{kPa}},\alpha_{p}$ = 21.4) of the tibialis anterior of rats (Bosboom et al. 2001). This model was chosen because as the fibers contract, it compresses the surrounding tissue, meaning that a model which describes the compressive properties of the tissue is needed.

### Numerical simulations

Comsol Multiphysics finite element solver (version 5.0, Cambridge, UK) was used to complete all simulations. This is a general-purpose solver, which allows access to the constitutive equations and the state variables in order to customize the problem set. In the simulations presented here, a fascicle of 19 muscle fibers (each surrounded by an endomysium sheet, see Fig. 1a, b) contracts as they are activated. A mesh of hexahedral (fiber) and quadrilateral (endomysium) elements with a Lagrange quadratic shape function was used. The mesh design for the model and the muscle fibers is shown in Fig. 1c, and specific values are in Table 4. The constitutive properties of the fibers and the connective tissue are those described in Sect. 2.1. In the model, the assumption was made that adjacent tissue connections could be modeled as fixed (tissue interfaces could not move relative to each other). From the simulations, transmission of predicted force from the active to the passive (non-activated) fibers occurred due to shear deformation through the endomysium. This was quantified by measuring the axial stress values in the model at a section in the middle of the fascicle across its axis (model stresses were obtained from the surface created from the plane described by the dashed line shown in Fig. 1c). In addition, the resulting axial stress and strain distribution of the whole muscle section values in the passive tissue were also quantified in the same plane to identify effects of fibers distribution and activation level in the surrounding tissue (Fig. 1 and Table 1).

**Table 4:** Mesh specifics of the fibers within the fascicle

Maximum element size	200 × thickness (aspect ratio 50)
Minimum element size	0.9092 µm (endomysium thickness (Zhang and Gao 2014))
Maximum element grow rate	2
Curvature factor	0.3
Resolution of narrow region	0.1

The fascicle containing 19 fibers was used to simulate different spatial distributions of activated fibers. It should be noted that the fascicle model excludes any tapering to the ends of the fibers as would be found in the real anatomy (Street 1983), but this was not the focus of this study. The active fibers represent slower fiber types (since they typically belong to smaller motor units that are thought to recruit denervated fibers during aging) and these were distributed either randomly across the area or, to represent age related reinnervation, clumped within a localized region of the fascicle cross section. Two cases of clumped active fibers (C1, C2) and two cases of sparse active fibers (S1, S2) were examined (see Figs. 3, 4 and Table 5 for activation patterns). In addition, activation level was manipulated by activating approximately 20% of the fascicle, 4 fibers (referred to as low level in subsequent text) or approximately 40% of the fascicle, 7 fibers (referred to as higher level in subsequent text) of the 19 fibers. These levels of activation were chosen to formulate case studies for the proof of concept proposed with this model. Specific activation levels and patterns within a fascicle are unknown, and this study has just chosen a range of activation patterns at two levels to demonstrate the concept of force transmission behavior. Finally, to verify how any changes in force transmission were influenced by more radical material properties, two further simulations were performed on case S1: one with a compliant (*E* = 5 kPa) and the other a stiffer (*E* = 30 MPa) extended tissue (tendon). These simulations were completed for one clumped, low activation level condition. Full details of activation pattern and level can be seen in Table 5.

**Table 5:** Total force transmitted to the passive fibers and stress in the connective tissue (young refers to the model with more compliant extended tissue, *E* = 5 kPa, and middle age refers to the model with stiffer extended tissue, *E* = 30 MPa)

	Configuration		Total transmitted force to passive fibers (mN)		Max. connective tissue axial stress (Pa)	
			Young	Middle Age	Young	Middle Age
Low-level activation	Clumped		0.123	0.123	3156	2702
			0.123	0.123	2402	2123
	Sparse		0.123	0.123	2431	1977
			0.123	0.123	4171	3510
Higher-level activation	Clumped		0.10	0.10	4012	3256
			0.10	0.10	8734	7517
	Sparse		0.10	0.10	4888	4079
			0.1	0.1	5223	4363

#### Mesh convergence and validation

A mesh convergence study was performed to verify the accuracy of the solution when simulating the contracting fascicle. To reduce the computational cost only a bundle of seven fibers was simulated using a symmetrical boundary condition. Convergence had occurred when 2716 elements were used, so this mesh design was used for all other simulations.

The simulation proposed is investigating the micromechanical behavior of the muscle tissue. At the moment, it is not possible to measure the activation pattern (micro-level) of the myofibers experimentally, but it is only possible to measure them as part of a whole muscle. For this reason, an analytical model based on Sharafi and Blemker’s (2010) using the MATLAB code provided by the paper adapted to study a single muscle fiber, surrounded by an endomysium sheet with 80% (aged) and 95% (young) volume fraction. The objective of running this analytical model is to verify whether the model proposed is a faithful representation of the muscle tissue. According to the analytical model, the force the endomysium exerted when shear deformation occurs is given by Eq. 10.

10 $$F_{\text{end}} = \frac{{2\left( {\frac{{G_{\text{end}} }}{{\sigma_{\text{iso}} }}} \right)\left( {\frac{{L_{0} }}{{r_{0} }}} \right)^{2} \lambda \left( {1 - \lambda } \right)}}{{ - 1 + \sqrt {1 + 2k\left( {2 + k} \right)\left( {\frac{\lambda }{\lambda + 1}} \right)} }},$$

where $\sigma_{\text{iso}}$ is the maximal isometric force, $L_{0}$ is the resting length, and $k$ is the ratio between the thickness and the endomysium thickness and the initial fiber radius. $G_{\text{end}}$ is the endomysium shear modulus, and since the Mooney–Rivlin model that represents the endomysium is not linear, the initial shear modulus was considered to be given by Eq. 11.

11 $$G_{\text{end}} = 2 \left( {c_{10} + c_{01} } \right),$$

where $c_{10}$ and $c_{01}$ are the first Mooney–Rivlin coefficients. (The initial shear modulus depends always on these first coefficients independently from the number of coefficients used for the model.) The results revealed that for fiber lengths greater than ten times the diameter, the force can be fully transmitted through the endomysium, while for the 80% volume fraction it must be for a ratio of 20. These ratios are far lower than physiological values that span between 300 and 4000 Sharafi and Blemker’s (2011) and further support the hypothesis of force transmission through the endomysium as it is interconnected along the full peripheral length of the fiber (Street 1983)which was also confirmed by the finite element model with linked domains proposed by Yucesoy et al. (2002). Hence, the analytical simulation verified that the constitutive properties chosen to model the bundle of fibers reflect the actual behavior of the muscle tissue, thus validating our model.

#### Boundary conditions

**Fig. 2:**
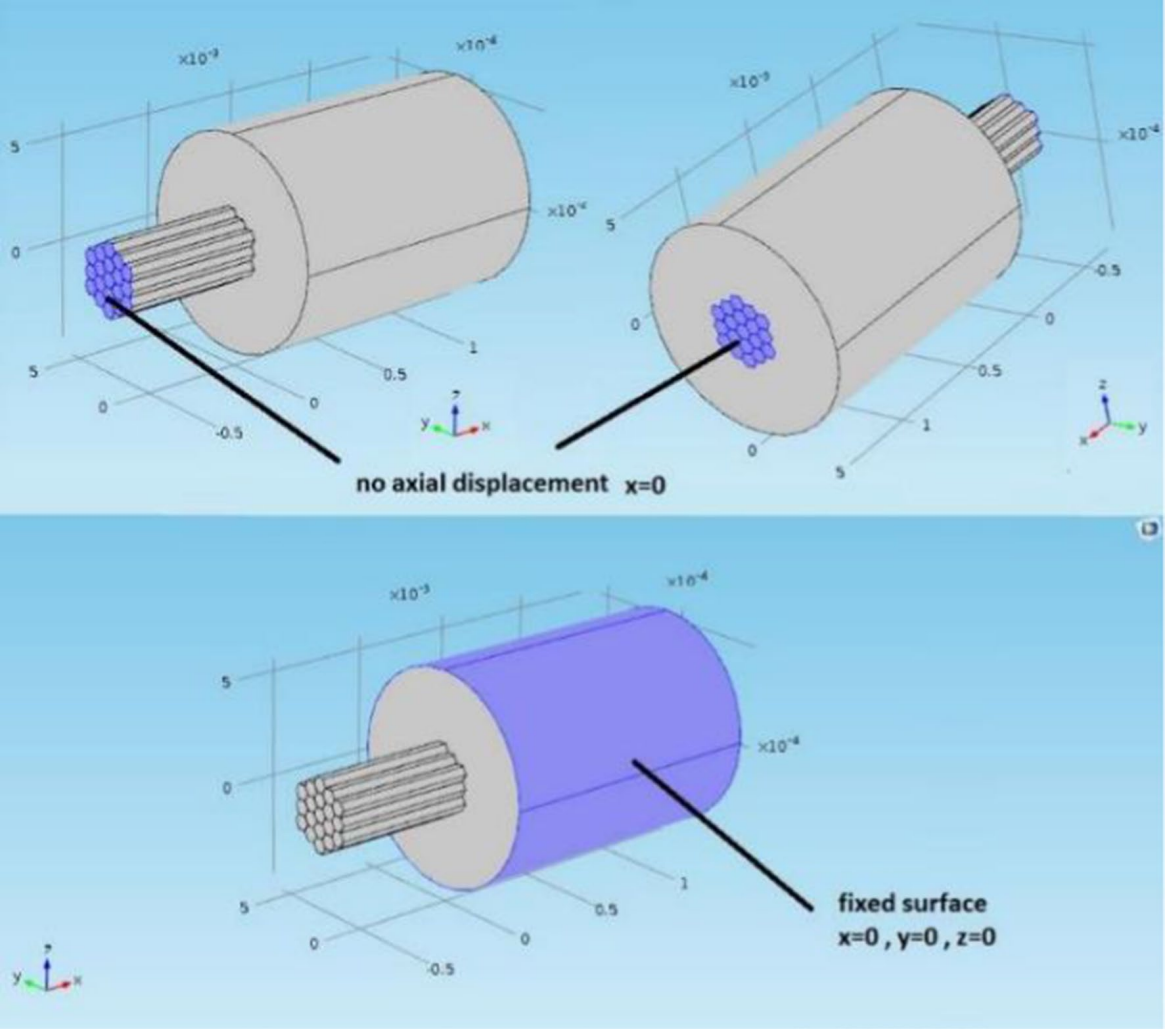
Boundary conditions of the FE model. The axial displacement is not permitted at the far ends, but other directions are permitted to allow the expansion of the fascicle as it is contracting. The outer layer of the surrounding tissue is fixed in all directions

The model was used to simulate an isometric contraction. The active stress was applied in the constitutive equations of the designated active fibers (as defined in Table 5). The displacement of the fascicle joined with the extended fascicle was constrained in the axial direction at each end; therefore, there was no overall axial length change of the combined tissues while the active fibers were contracting. The outer circumference of the surrounding inactive muscle tissue domain was fixed in all degrees of freedom to represent the inertia of the whole muscle (see Fig. 2).

#### Processing

The total force transmitted to the passive fibers was computed through the finite element model for each distribution and activation level case. The force was calculated as the average axial stress in the passive fiber (at the clamped end of the fascicle) times the passive fiber area. The average stress was obtained by setting area probes at each surface of the passive fibers. In addition, the axial strain/stress (see Fig. 1c—dotted line made a plane that cut through the model) distribution was evaluated at a cross section in the middle of the fiber. A custom written MATLAB m file (R2013a, Mathworks, US) was used to analyze and compare the resulting data.

## Results

### Force transmission between active and passive fibers

From results shown in Table 4, there was no difference in the total axial force transmitted to the passive fibers (of the fascicle) with different activation patterns across the fascicle containing 19 fibers. In the case of higher-level activation, the results were still equivalent, although the total axial force recorded in the passive fibers was lower as there were fewer passive fibers (Table 5). The simulations incorporating more compliant or stiffer extended tissue also predicted, respectively, 0.11 mN and 0.124 mN force transmission which proves no apparent effect of these properties on lateral force transmission to the passive fibers as predicted in Table 5.

### Stress and strain distributions across the simulated fascicle

**Fig. 3:**
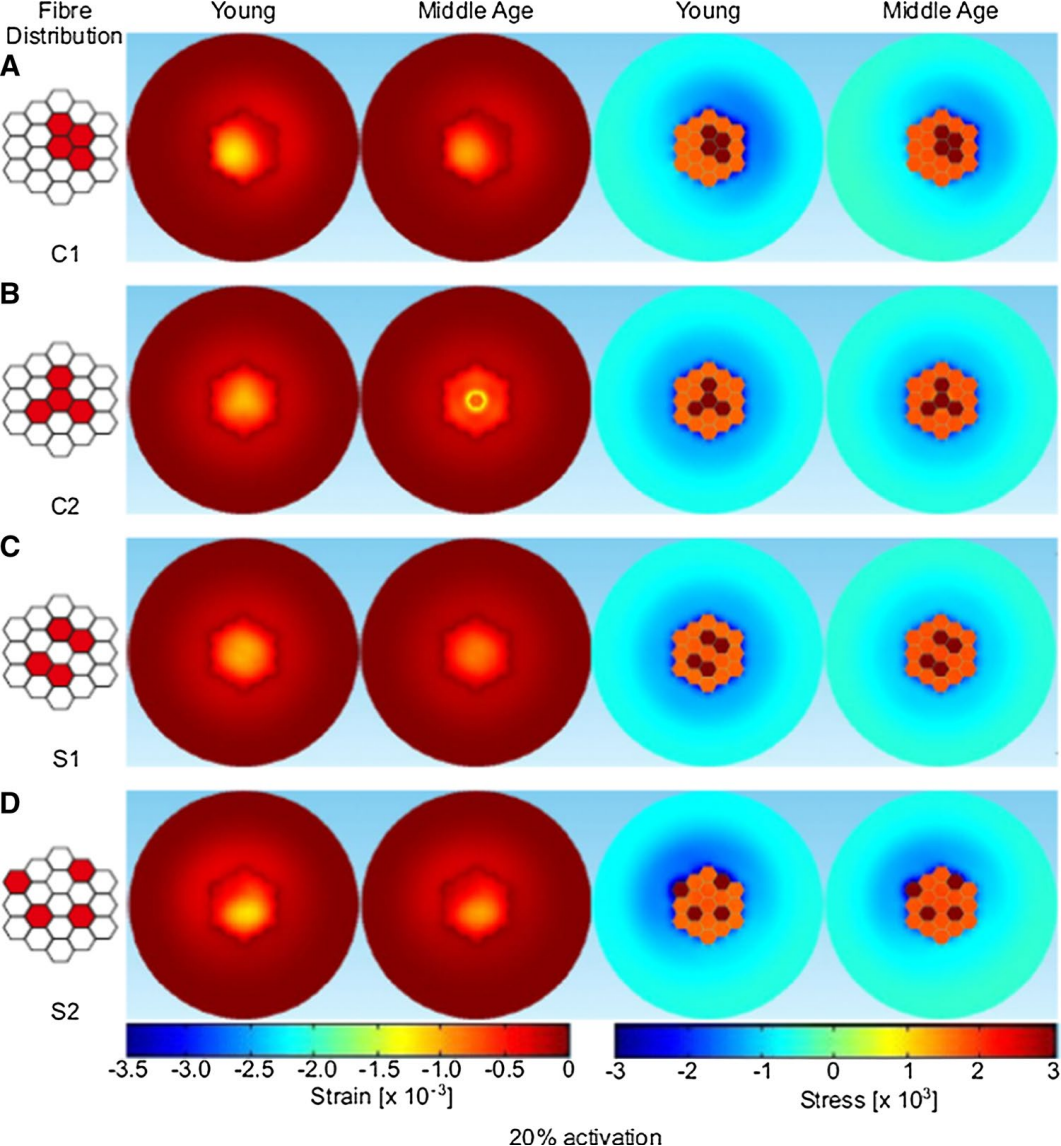
Distribution of strain and stress values predicted through the fascicle cross section, for simulations of young (more compliant extended tissue) and middle age (stiffer extended tissue) tissue for the low activation cases (20% activation). Distribution of the active fibers are shown on the left

**Fig. 4:**
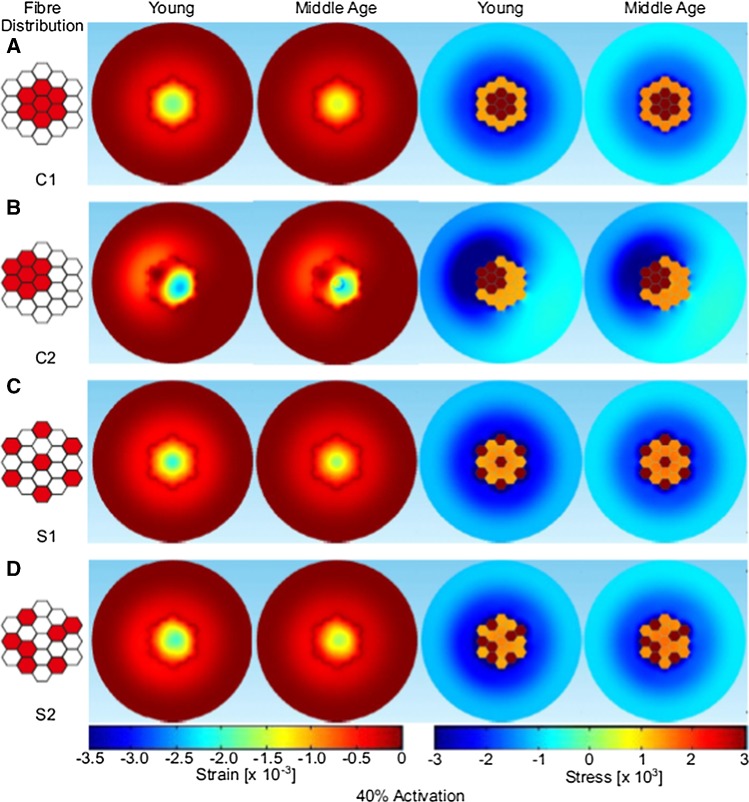
Distribution of strain and stress values predicted through the fascicle cross section, for simulations of young (more compliant extended tissue) and middle age (stiffer extended tissue) tissue for the higher activation cases (40% activation). Distribution of the active fibers are shown one the left

A cross section (radial plane) in the middle of the fascicle was considered to explore the distribution of the stress and strains. Figures 3 and 4 show that the axial stress distribution within the fascicle is evenly distributed across the passive fibers. In contrast the axial stress in the inactive muscle tissue surrounding the fascicle is more affected by the activation level and fiber distribution. Specifically, when the active fibers are at the edge of the fascicle, the axial stress distribution in the inactive muscle tissue becomes strongly uneven as can be seen in the lower activation level for C1 and S2 patterns (Fig. 3). For the higher-level activation (Fig. 4), C2 activation pattern has a higher axial stress concentrated in the region around the active fibers. In this case, the clumped active fibers are located at the edge of the fascicle and the induced stress in the inactive muscle tissue region is not only considerably higher than the opposite area (bottom right), but it has a maximum stress (− 8734 Pa) which is almost double compared to the maximum axial stress seen in the other activation patterns. The effects of the location of the clumped fiber patterns can also be seen in the axial stress and strain patterns observed in the middle age condition, although it should be noted the axial stress and strain values are lower than those from the simulations using more compliant tissue properties (i.e., younger tissue) (Figs. 3 and 4). For the low-level activation, the axial strain is greater toward the middle of the fascicle away from the interface with the surrounding tissue which is stiffer and has a bigger dimension (Fig. 3). Similar results were found for the higher-level activation (Fig. 3), with higher value of axial strain due to the fact that more fibers are activated.

## Discussion

In this paper, a finite element method approach was applied to study the micromechanical properties of skeletal muscle tissue, with a focus on investigating the influence of the level of activation, the distribution of activated fibers and the stiffness of material properties on force transmission and patterns of stress and strain distribution. A contracting fascicle model was implemented, and the results suggest no small changes in the axial force transmitted to the passive fibers within the fascicle tissue due to variation in the activation distributions (see Table 4, first two columns). The actual force transmitted to the passive fibers remains 0.123 mN for low-level activation and 0.1 mN for higher-level activation (see Table 5). The location and distribution of activated fibers in the fascicle tissue did, however, influence patterns of axial stress and strain observed in the surrounding inactive muscle tissue, particularly when active patterns involved fibers that were clustered and closer to the edge of the fascicle (see Table 5). For example, C2 of higher-level activation produced the maximum stress in the connective tissue which was concentrated on one side (the part where the active fibers are clumped at the edge, see Table 5 and Fig. 4).

The fiber activation patterns studied here could be argued to not represent normal physiological conditions, as fibers belonging to the same motor unit are typically described as being randomly distributed across the muscle cross section (Vieira et al. 2016). However, fiber clustering does occur in aged muscle (Faulkner et al. 2007; Lexell et al. 1988) where it is the consequence of axonal sprouting from motor units to innervate nearby denervated fibers (Brown et al. 1981). Greising et al. (2015) processed histological data of mice diaphragm muscle to identify fibers types, the presence of sarcopenia and clustering of fibers. As expected, the sarcopenic condition was confirmed by a loss of type IIx/IIb type fibers. In addition, the clustering of fibers was identified and quantified by calculating the distance of a fiber, from surrounding fibers of the same type (closest distance). The clustering of type IIa fibers was confirmed by the higher proportion in old mice (since they reinnervated type IIx/IIb), whereas the clustering of type I fibers was assessed by a decreased distance between fibers of the same type. The macromechanical consequence of this remodeling is a decrease in the total muscle force (Faulkner et al. 2007; Kadhiresan et al. 1996); however, the consequences on a micromechanical level have not previously been reported. The results from our paper show the consequences on a micromechanical level.

The results from the simulations presented in our paper show no difference in the transmitted force between the different fiber distributions studied. This suggests that clustering does not lead to decreased force transmission. However, there was an effect on the patterns of stress and strain distributions observed (Figs. 3, 4). The axial strain patterns revealed that in the fascicle the strain is higher in regions far from the interface with the surrounding inactive muscle tissue. When the active fibers are packed close to the interface, higher strains are observed on the opposite side. This likely reflects the effects of differences in the stiffness of the fascicles and the surrounding materials. This is exacerbated when the active fibers are localized at the edge of the fascicle as two regions with different tissue stiffness are created, one being the stiffer active fibers and the other the more compliant passive fibers. The potential effect of this is an unbalanced distribution of the stress. It should be considered that damage could occur at the interface between the fascicle and the surrounding tissue since the transmission of the force is concentrated on one side. As such unbalanced stress distribution could cause damage of the fascicle during dynamic and/or high loading conditions. These excessive strains could lead to Sarcomere popping which was suggested to be a possible consequences by Yucesoy et al. (2002). Yucesoy also produced a finite element model with a linked fiber-matrix mesh to study the interaction between the muscle fibers and the extracellular matrix. This allowed a direct investigation of the effect of a disruption of the links between these two domains. Models of a pennate muscle with stiff and compliant fiber-matrix links were simulated. The compliant links led to excessive strains and obtained similar conclusions to our study. In the work reported here the compliant tissue can be seen as the tissue with no active fibers when clustering occurs since there are regions where the tissue deformation is higher.

In the performed simulation, the strains were small since only an isometric contraction was considered and the stress transmitted to the passive fibers was only 2% of the maximal isometric stress. Therefore, we propose that motor unit remodeling associated with aging (activation patterns) does not only decrease force producing capacity as reported in the literature (Lexell et al. 1988), but could lead also to unbalanced strains and damage to tissues at higher strain levels which may occur during higher levels of activation and/or during dynamic conditions.

When changing the stiffness of the extended tissue to mimic those seen at different ages, no changes occurred to the force transmitted (Table 5), probably due to the low magnitude strain associated with the isometric contraction. A small increase in the strain seems to occur in the simulation of young tissue and can be justified by the fact that the extended tissue is more compliant. This has the effect of increasing the stress throughout the tissue, a finding that is in line with Sharafi et al. (2011), who showed that peak strains occur when the endomysium is more compliant, and that these peak strains are located near the muscular–tendon junction. In this study, a compliant tissue results in a more even distribution of the stress throughout the whole tissue, but further studies are needed to more fully explain the macromechanical consequences. Rahemi et al. (2015) produced a macromechanical model which considered fat infiltration. Fat tissue is stiffer than muscle tissue, and this resulted in a resistance to the fiber contraction which led to a lower force transmission, in line with our results. The downside of higher strains and therefore stresses was shown to be a risk of injuries when the muscle is lengthening in Sharafi and Blemker (2011) validated model.

Our study considers the micromechanical behavior of the muscle at a fiber/fascicle level. It highlighted how clustering of fibers can induce stress concentrations that may be harmful at higher strains. At a fascicle level, the perimysium not only transmits the stress but must allow higher strains to accommodate fascicle shape changes (Purslow 2002; Meyer and Lieber 2011). Sharafi and Blemker (2010) showed fascicles have variable geometry that fit the muscle shape and mechanical function. In our study, the unbalanced stress within the fascicle also induces an uneven stress outside the fascicle, specifically when clumped activated fibers are close to the edge of the fascicle. This can compromise the ideal stress and strain distribution across the muscle which is facilitated by the fascicle geometry. Further work using a joint experimental and simulation study approach could be used to explain possible alterations in fascicle geometry of older muscle tissue or clarify factors underpinning localized higher stress and/or strain.

The fascicle modeled represents only a portion of the total fascicle length in the real anatomy and that motivated the addition of a domain as an extension to represent the tendon. Considering only a portion of the whole fascicle is a limitation to this work, but this approach limits computational time and the complexity of having to consider other fiber morphological features such as tapering at the ends (Monti et al. 1999). Effects of an unbalanced partial activation of the muscle on the full length of the fiber can be derived from work by Turkoglu et al. (2014) where they show a simulation of a partially paralyzed (non-active) muscle. Turkoglu et al. (2014) used a FE simulation, which activated only a region of the muscle (proximal, middle, distal) and as a consequence the activated fibers were forced to be at longer sarcomere lengths by the paralyzed fibers. Their model also proved that an increased stiffness of the ECM further enhances this mechanism (Turkoglu and Yucesoy 2016). In relation to the result from the micromechanical model of shown in our paper, it could be argued that a sparse activation would create a uniform distribution of the stresses. This can explain the results from experimental studies (Monti et al. 2001; Vieira et al. 2011) where while a motor unit can be localized in a defined region, the fibers belonging to the motor unit were randomly distributed within that region. This distribution is altered in aged muscle, specifically slow twitch motor units (Ansved et al. 1991) which recruit new fibers, leading to clusters of activated fibers adjacent to non-active fibers, which may cause an alteration of the muscle properties. The implication of a cluster activation verses a sparse activation should be tested experimentally, although it is not possible to selectively activate a single fiber within a fascicle at a micromechanical level, meaning that models such as the one in our study provide insightful indications for possible biomechanical behavior. The alteration is likely to be disruptive since limiting the functionality of the muscle [e.g., lower range of motion (Turkoglu et al. 2014)] and introducing higher stresses/deformations as confirmed our model shown in this paper. Additionally our study did not consider pennation angle, as this was out of the scope of this study, but it is likely that the effects would be reduced by a proportion equal to the cosine of the angle of pennation, but to draw firm conclusions further work would be needed to evidence this. Future studies should take into consideration the portion of fibers at the muscular–tendon junction, where tapering of the fibers should be considered to enable fuller evaluation of features of activation level and fiber clustering across the whole muscle tendon unit.

## Conclusion

The spatial clustering of muscle fibers within the same motor unit is one of the consequences of motor unit loss and reinnervation associated with aging. In this study, isometric contractions of a fascicle containing 19 fibers were simulated at different activation levels and with different spatial distributions of activated fibers. The results did not reveal evident quantitative changes in force transmission, but the patterns of stress and strain distribution were affected, suggesting an uneven distribution of the forces may occur within the fascicle that may cause damage for higher levels of strain. This phenomenon could explain the mechanism for muscle injury in older muscle.
